# A call for inclusion of arthroplasty as essential surgery

**DOI:** 10.2471/BLT.25.294300

**Published:** 2026-02-03

**Authors:** Peter G Delaney, Nicolas S Piuzzi

**Affiliations:** a Department of Orthopaedic Surgery, Orthopaedic & Rheumatologic Institute, Cleveland Clinic Foundation, 9500 Euclid Avenue Suite A40, Cleveland, Ohio, 44195, United States of America.

## Abstract

In 2015, the World Health Assembly and the *Lancet* Commission on Global Surgery affirmed the need for equitable access to essential surgical care. Additionally, the World Bank’s Disease Control Priorities project identified 44 essential surgical procedures for health-care systems in low- and middle-income countries. These procedures address the global burden of disease, are cost-effective and are feasible to implement in resource-constrained settings. Notably, arthroplasty, specifically, hip and knee replacements, was excluded. A decade later, this omission warrants reconsideration. With improved control of communicable diseases, musculoskeletal conditions are now the second leading cause of years lived with disability globally, disproportionately affecting populations of low- and middle-income countries. Arthroplasty meets all the criteria for an essential surgical procedure: it is cost-effective, yields predictable and durable outcomes, and follows standardized clinical pathways suited for large-scale implementation. The cost of arthroplasty per disability-adjusted life year averted is often lower than treatments for chronic medical conditions and communicable diseases. Rates of dislocation and periprosthetic joint infection for arthroplasty in sub-Saharan Africa were comparable to rates in high-income settings, suggesting feasibility when appropriate systems are in place. As life expectancy and the noncommunicable disease burden increase, functional mobility interventions and pain relief will become a public health priority. Integrating arthroplasty into national surgical plans is essential to build resilient surgical systems that respond to evolving demographic and epidemiological trends. We call for the formal recognition of arthroplasty as an essential surgical procedure and for investment in workforce training, supply-chain infrastructure and funding models to meet unmet global surgical needs.

## Résumé

En 2015, l’Assemblée mondiale de la Santé et la Commission du Lancet sur la chirurgie mondiale ont affirmé la nécessité d’un accès équitable aux soins chirurgicaux essentiels. En outre, le projet « Disease Control Priorities » (Priorités en matière de lutte contre les maladies) de la Banque mondiale a identifié 44 interventions chirurgicales essentielles pour les systèmes de santé des pays à revenu faible et intermédiaire. Celles-ci permettent de lutter contre la charge mondiale de morbidité, sont rentables et peuvent être mises en œuvre dans des contextes de ressources limitées. Il convient de noter que l’arthroplastie​, et plus spécifiquement ​l’arthroplastie​ de la ​h​anche et ​du genou,​​ en a été exclue. Une décennie plus tard, cette omission mérite un réexamen. Grâce à l’amélioration de la lutte contre les maladies transmissibles, les troubles musculo-squelettiques représentent désormais la deuxième cause mondiale d’années vécues avec un handicap. Ils concernent de manière disproportionnée les populations des pays à revenu faible et intermédiaire. L’arthroplastie répond à tous les critères d’une intervention chirurgicale essentielle: elle est rentable, fournit des résultats prévisibles et durables et applique des protocoles cliniques standardisés adaptés à une mise en œuvre à grande échelle. Le coût de l’arthroplastie par année de vie ajustée sur l’incapacité évitée est souvent inférieur à celui du traitement des maladies chroniques et des maladies transmissibles. Les taux de luxation et d’infection articulaire périprothétique pour l’arthroplastie en Afrique subsaharienne étaient comparables à ceux observés dans les pays à revenu élevé, ce qui suggère la praticabilité de cette intervention lorsque les systèmes appropriés sont en place. À mesure qu’augmentent l’espérance de vie et la charge de morbidité des maladies non transmissibles, les interventions visant à améliorer la mobilité fonctionnelle et à soulager la douleur deviendront une priorité de santé publique. L’intégration de l’arthroplastie aux plans nationaux d’interventions chirurgicales est capitale pour mettre en place des systèmes chirurgicaux résilients qui suivent l’évolution des tendances démographiques et épidémiologiques. Nous appelons à la reconnaissance officielle de l’arthroplastie comme intervention chirurgicale essentielle et à l’investissement dans la formation de la main-d’œuvre, les infrastructures de la chaîne d’approvisionnement et les modèles de financement afin de répondre aux besoins chirurgicaux non satisfaits à l’échelle mondiale.

## Resumen

En 2015, la Asamblea Mundial de la Salud y la Comisión Lancet sobre Cirugía Global reafirmaron la necesidad de un acceso equitativo a la atención quirúrgica esencial. Además, el proyecto Disease Control Priorities del Banco Mundial identificó 44 procedimientos quirúrgicos esenciales para los sistemas de salud en países de ingresos bajos y medios. Estos procedimientos abordan la carga mundial de morbilidad, son costoefectivos y son factibles de implementar en entornos con recursos limitados. Cabe destacar que la artroplastia, específicamente reemplazos de cadera y rodilla, fue excluida. Una década después, esta omisión merece ser reconsiderada. Con el mejor control de las enfermedades transmisibles, las afecciones musculoesqueléticas constituyen ahora la segunda causa mundial de años vividos con discapacidad, y afectan de manera desproporcionada a las poblaciones de países de ingresos bajos y medios. La artroplastia cumple todos los criterios de un procedimiento quirúrgico esencial: es costoefectiva, produce resultados previsibles y duraderos, y sigue vías clínicas estandarizadas adecuadas para la implementación a gran escala. El costo de la artroplastia por año de vida ajustado por discapacidad evitado suele ser inferior al de tratamientos para enfermedades crónicas y enfermedades transmisibles. Las tasas de luxación e infección articular periprotésica asociadas a la artroplastia en el África subsahariana fueron comparables a las observadas en entornos de ingresos altos, lo que sugiere viabilidad cuando existen sistemas adecuados. A medida que aumenten la esperanza de vida y la carga de enfermedades no transmisibles, las intervenciones para mejorar la movilidad funcional y el alivio del dolor se convertirán en una prioridad de salud pública. Integrar la artroplastia en los planes quirúrgicos nacionales es esencial para construir sistemas quirúrgicos resilientes que respondan a las tendencias demográficas y epidemiológicas cambiantes. Se insta al reconocimiento formal de la artroplastia como procedimiento quirúrgico esencial y a invertir en la formación del personal, la infraestructura de la cadena de suministro y modelos de financiación para atender las necesidades quirúrgicas mundiales no cubiertas.

## ​​ملخص​

​​في عام 2015، أكدت كل من جمعية الصحة العالمية، ولجنة لانسيت للجراحة العالمية، الحاجة إلى الحصول العادل على الرعاية الجراحية الأساسية. بالإضافة إلى ذلك، فإن مشروع أولويات مكافحة الأمراض التابع للبنك الدولي، قام بتحديد 44 إجراءً جراحيًا أساسيًا لأنظمة الرعاية الصحية في الدول ذات الدخل المنخفض والدول ذات الدخل المتوسط. تتناول هذه الإجراءات العبء العالمي للأمراض، وهي فعّالة من حيث التكلفة وقابلة للتنفيذ في البيئات محدودة الموارد. ومن اللافت أن جراحة ​​استبدال​​ ​​المفصل​​، ​​وعلى​​ ​​وجه​​ ​​التحديد​​ ​​عمليات​​ ​​استبدال​​ ​​الورك​​ ​​والركبة​​،​​​​ ​​ لم تُدرج ضمنها. وبعد عقد من الزمن، يستحق هذا الاستبعاد إعادة النظر. فمع تحسن السيطرة على الأمراض المعدية، أصبحت أمراض الجهاز العضلي الهيكلي ثاني أكبر سبب عالميًا لسنوات الحياة بالإعاقة، وهي تؤثر بشكل غير متناسب على سكان الدول منخفضة الدخل ومتوسطة الدخل. تستوفي جراحة استبدال المفصل جميع معايير الإجراء الجراحي الأساسي: فهي فعّالة من حيث التكلفة، وتحقق نتائج متوقعة ومستدامة، وتتبع مسارات إكلينيكية موحدة مناسبة للتنفيذ على نطاق واسع. غالبًا ما تكون تكلفة استبدال المفصل لكل سنة يتم تجنبها من معدلات سنوات العمر المعدلة بالإعاقة، أقل من تكلفة علاج الحالات الطبية المزمنة والأمراض المعدية. كما أن معدلات الخلع والعدوى حول المفصل الصناعي في جنوب الصحراء الكبرى كانت مماثلة للمعدلات في البيئات ذات الدخل المرتفع، مما يشير إلى إمكانية التنفيذ عند توفر الأنظمة المناسبة. ومع زيادة متوسط العمر المتوقع وعبء الأمراض غير المعدية، ستصبح التدخلات التي تعزز الحركة الوظيفية وتخفيف الألم، أولوية للصحة العامة. إن دمج جراحة استبدال المفصل في الخطط الجراحية الوطنية يعد أمرًا ضروريًا لبناء أنظمة جراحية مرنة تستجيب للتغيرات السكانية والوبائية المتطورة. ندعو إلى الاعتراف الرسمي بجراحة استبدال المفصل كإجراء جراحي أساسي، وإلى الاستثمار في تدريب القوى العاملة، وبنية تحتية لسلاسل الإمداد، ونماذج تمويل لتلبية الاحتياجات الجراحية العالمية غير المستوفاة.​

## 摘要

世界卫生大会和《柳叶刀》全球外科委员会均于 2015 年强调了实现基本外科护理服务公平获取的必要性。此外，世界银行的疾病控制优先事项项目确定了应被纳入中低收入国家卫生保健系统的 44 种基本外科手术。此类手术有助于减轻全球疾病负担，具有较高的成本效益，且能够在资源有限地区得以实施。值得注意的是，关节置换术​（特别是髋关节和膝关节置换术）​并未被纳入此类手术范畴。在十年后的今天，我们有必要重新考虑这种被忽略的手术。随着传染病已得到更有效的控制，肌肉骨骼疾病现已成为导致全球伤残损失寿命年的第二大主要原因，并对中低收入国家的人口造成了不成比例的影响。关节置换术符合基本外科手术的所有相关标准：具有较高的成本效益、能产生可预测的持久疗效以及遵循适合大规模实施的标准化临床路径。如果基于每个可避免的伤残调整生命年进行计算，则关节置换术的成本通常要低于慢性疾病及传染病的治疗成本。在撒哈拉以南非洲地区，关节置换术后出现脱位和假体周围感染的比率与高收入国家/地区相当，这表明只要构建适当的系统，即可实施此类手术。随着预期寿命的延长以及非传染性疾病负担的加重，提高功能性活动能力和缓解疼痛将会成为公共卫生系统需优先解决的问题。将关节置换术纳入国家外科手术计划，对于构建具有韧性的足以应对不断变化的人口和流行病学发展趋势的外科手术系统而言至关重要。我们呼吁将关节置换术正式纳入基本外科手术范畴，并呼吁投资于人力培训、供应链基础设施及筹资模式，以填补全球的外科手术需求缺口。

## Резюме

В 2015 году Всемирная ассамблея здравоохранения и Комиссия издания Lancet по глобальной хирургии подтвердили необходимость справедливого доступа к основным хирургическим услугам. Кроме того, проектом Всемирного банка по приоритетам контроля заболеваний (Disease Control Priorities) были определены 44 основные хирургические процедуры для систем здравоохранения в странах с низким и средним уровнями дохода. Эти процедуры решают проблемы глобального бремени заболеваний, экономически эффективны, и их можно осуществлять в условиях ограниченных ресурсов. Следует отметить, ​​что из этого списка была исключена артропластика, а именно эндопротезирование тазобедренного и коленного суставов​​​​ ​​. Десять лет спустя нам стоит пересмотреть это решение. На фоне улучшения контроля над инфекционными заболеваниями второй по распространенности ведущей причиной жизни с инвалидностью во всем мире стали заболевания опорно-двигательного аппарата, при этом доля жителей стран с низким и средним уровнем дохода среди таких больных непропорционально велика. Артропластика соответствует всем критериям основной хирургической процедуры: она экономична, дает предсказуемые и долговременные результаты, при ее проведении соблюдаются стандартизированные клинические протоколы, что удобно для масштабного внедрения. Затраты на артропластику в расчете на один предотвращенный год жизни с поправкой на инвалидность часто ниже, чем стоимость лечения хронических соматических состояний и инфекционных заболеваний. Частота вывихов и перипротезной инфекции суставов при артропластике в странах Африки южнее Сахары была сопоставима с показателями для стран с высоким уровнем дохода, что говорит о практической реализуемости таких вмешательств при наличии соответствующих систем. По мере увеличения ожидаемой продолжительности жизни и бремени неинфекционных заболеваний вмешательства, направленные на сохранение функциональной мобильности и обезболивание, станут приоритетными в общественном здравоохранении. Введение артропластики в национальные хирургические планы крайне важно для построения устойчивых и адаптивных хирургических систем, способных реагировать на изменяющиеся демографические и эпидемиологические тенденции. Авторы призывают официально признать артропластику необходимой хирургической процедурой и вкладывать средства и усилия в обучение персонала, инфраструктуру цепочки поставок и модели финансирования для как можно более полного удовлетворения глобальных хирургических потребностей.

## Introduction

In 2015, the World Health Assembly passed a resolution committing to strengthening emergency and essential surgical care and anaesthesia globally (WHA68.15).^1^ At the same time, the *Lancet* Commission on Global Surgery published a set of guidelines for achieving equitable surgical delivery within the context of universal health coverage (UHC).^2^ Supporting the prioritization of surgical capacity-building efforts globally, the *Lancet* Commission guidelines focused on access to timely and safe surgery, increasing surgical workforce density, increasing surgical volume in low- and middle-income countries, reducing perioperative mortality, and protecting against impoverishing and catastrophic health expenditure.^3^ The third edition of the World Bank Disease Control Priorities project subsequently defined 44 essential surgical procedures as a starting point for health-care systems in low- and middle-income countries.^4^ Essential surgical procedures are defined as interventions that address a substantial need that can be successfully treated by surgery, are cost-effective and are feasible to implement globally.^5^ Although the authors acknowledged the list was not exhaustive, essential orthopaedic surgical procedures were mainly injury-related, given that a disproportionate share of the global trauma burden occurs in low- and middle-income countries. These procedures include non-displaced fracture management, fracture reduction, irrigation and debridement of open fractures, placement of external fixators, use of traction, fasciotomy and trauma-related amputations. The only non-trauma orthopaedic essential surgical procedures are for acute conditions threatening life or limb such as drainage of septic arthritis and debridement of osteomyelitis. All these procedures were designated to be performed at local hospitals, implying the availability of adequately developed surgical capabilities and doctors with surgical expertise and the need for only basic universal surgical instruments, with the exception of external fixation and traction supplies. The Disease Control Priorities project acknowledged that other procedures for trauma care would need to be performed at higher-level facilities with advanced expertise, including in vascular repair, open reduction and internal fixation, drainage of intracranial haematoma, and exploration of the neck or chest.^4^ However, arthroplasty, specifically, hip and knee replacements, was omitted from the list of essential surgical procedures.

In the decade since WHA 68.15 and the *Lancet* Commission on Global Surgery recommendations, national surgical, obstetric and anaesthesia plans have been developed in several countries to direct surgical capacity-building efforts.^6^ However, only 26.3% (21/80) of low- and middle-income countries are on track to meet the 2030 access targets, 41.1% (60/146) for the workforce target, and none for the surgical volume target.^7^ As such, there is an unmet need of at least 160 million operations annually based on the *Lancet* Commission benchmark of 5000 procedures per 100 000 population annually.^7^ Although seven multinational studies and 10 national studies reported access across 96 countries, the six *Lancet* Commission surgical indicators: (i) access; (ii) workforce; (iii) operative volume; (iv) postoperative mortality; (v) protection against impoverishing expenditure; and (vi) protection against catastrophic expenditure, have not been consistently reported by governments.^7^ Without global monitoring, the creation of a global action plan to ensure that the *Lancet* Commission targets are met will not be feasible. Although modelled projections for disparities in surgical access have not been updated since the *Lancet* Commission’s landmark publication in 2015, it is likely that no reduction has occurred in the 4.8 billion people lacking access to safe surgery.^7^

Given evolving global demand and the need for an update on the list of essential surgical procedures after 10 years, the addition of arthroplasty should be considered, especially as admissions for musculoskeletal disorders represent the largest proportion of patients requiring some type of surgical procedure. Based on the Disease Control Priorities definition that essential surgical procedures address a global burden of disease, are cost-effective and are feasible to implement even in resource-constrained settings, we aim to show that arthroplasty meets each of these criteria and is a fundamental surgical offering that should be prioritized by policy-makers alongside ongoing global surgical capacity-building efforts.

## Global burden of disease

While the global health sector has traditionally prioritized communicable diseases such as human immunodeficiency virus (HIV), tuberculosis and malaria, noncommunicable diseases are now the leading cause of death and disability globally, and this disease burden is disproportionately borne by low- and middle-income countries.^8^ With improved control of communicable diseases, life expectancy in resource-constrained settings has risen significantly, for example, from 52.7 years in 2000 to 66.1 years in 2021 in sub-Saharan Africa.^9^ As a result, an ageing population is increasingly affected by disability from noncommunicable diseases, including musculoskeletal conditions. Acquired musculoskeletal disorders, such as degenerative joint diseases, are now the second leading cause of years lived with disability (YLDs): osteoarthritis accounts for 2.3% (95% uncertainty interval, UI: 1.4–4.5) of all-cause YLDs, while other musculoskeletal disorders account for 4.8% (95% UI: 3.6–6.2). Only low back pain accounts for more, at 7.7% of all-cause YLDs (95% UI: 6.5–9.1).^4^^,^^10^ In 2020, 595 million people (95% confidence interval, CI: 535 million–656 million) had osteoarthritis, equating to 7.6% (95% CI: 6.8–8.4) of the global population. This figure is projected to increase by 74.9% (95% CI: 59.4–89.9) for knee and 78.6% (95% CI: 57.7–105.3%) for hip osteoarthritis to nearly 1 billion individuals by 2050.^11^

For patients with end-stage osteoarthritis, hip and knee arthroplasty remains the most effective intervention to restore function and mobility, yet it is still under-recognized in global health planning. This misalignment is not limited to low- and middle-income countries but also affects high-income countries. For example, in the United States of America in 2019, musculoskeletal conditions affected 127.4 million people and they were the top driver of health-care spending in 2016 (380.9 billion United States dollars, US$), yet they receive disproportionately low research funding from the National Institutes of Health.^12^ Additionally, the global incidence of hip fractures was estimated to exceed 10 million cases in 2018 and is expected to nearly double by 2050.^13^ Even though perioperative delays in hip fracture care longer than 24 hours are associated with significantly higher risks of 30-day mortality, pulmonary embolism, deep venous thrombosis and myocardial infarction, arthroplasty, which is an established treatment for displaced femoral neck fractures, has not been prioritized as it is often viewed as an elective surgical offering.^14^ The current spectrum of care for osteoarthritis includes non-pharmacological, pharmacological and surgical management, although no disease-modifying treatments exist. First-line management comprises education, exercise and weight management, and non-steroidal anti-inflammatory drugs.^15^ Intra-articular corticosteroid injections are frequently used as a subsequent palliative measure to provide relief, although studies have called into question the efficacy of such treatment. For example, a Cochrane systematic review found low-quality evidence of a small to moderate benefit by 6 weeks that was not sustained.^16^ When conservative measures fail to prevent degenerative progression, arthroplasty is indicated to maintain movement and quality of life. Although non-arthroplasty surgical options exist, such as high tibial osteotomies to offload arthritic medial knee compartments, and peri-acetabular osteotomies or valgus-producing femoral osteotomies for cases of hip dysplasia to slow degenerative changes and delay arthroplasty, the definitive solution for end-stage osteoarthritis is arthroplasty.

Significant gaps in access to specialists exist in low- and middle-income countries. Therefore, conservative alternatives for osteoarthritis management should be sought and implemented in the short-term. At the same time, global surgical capacity-building efforts should be undertaken to incorporate arthroplasty planning. Currently, surgical conditions are estimated to account for about a third of the global burden of disease. However, around 5 billion people globally lack access to surgery and 1.5 million people die each year from a lack of surgical interventions. Furthermore, an additional 160 million surgical procedures are required annually to meet the current unmet total surgical need.^2^^,^^5^^,^^17^^,^^18^

## Cost–effectiveness

The notion that surgery is too complicated or expensive to implement as a public health measure is changing, as surgery has been found to be cost-effective compared with other public health interventions. However, cost–effectiveness varies widely depending on the intervention performed. When measured using cost per disability-adjusted life year (DALY) averted, cost–effectiveness has been estimated at US$ 10.93 per DALY averted for emergency obstetric care at a rural hospital in Bangladesh, US$ 32.78 per DALY averted for all surgical care services in a Sierra Leonean hospital and US$ 315.12 per DALY averted for caesarean deliveries.^19^^–^^21^ In 2014, a systematic review of the cost–effectiveness of surgical interventions in low- and middle-income countries generated a composite cost–effectiveness ratio for orthopaedic surgery using data from different orthopaedic surgery procedures performed in Dominican Republic, Haiti and Nicaragua. These procedures included surgeries for congenital malformations, acute fractures, chronic mal-unions and non-unions, and osteomyelitis with the cost equating to US$ 381.15 per DALY averted.^21^ Despite the surgical infrastructure requirements, orthopaedic procedures demonstrated value especially when compared with medical treatment for chronic ischaemic heart disease (US$ 500.41–706.54 per DALY averted) and HIV treatment (US$ 453.74–648.20 per DALY averted), as surgical patients are less likely to be under continuous monitoring for their disease in contrast to communicable diseases.^21^ The literature on orthopaedic cost–effectiveness analyses in low- and middle-income countries is sparse. Without robust cost–effectiveness data from such settings to generate cost–effectiveness ratios, other than from the Central American and Caribbean study, policy-makers must consider setting-specific factors when assessing local relevance. Alternatively, outcomes from similar settings may provide relevant experience for future cost projections. Malawi, for instance, which is the only sub-Saharan African country with a national joints registry, published its first 10-year outcomes of primary total hip arthroplasty, which provides a benchmark against which policy-makers in other settings may extrapolate or compare results.^22^

Elective primary hip and knee arthroplasty also leads to substantially increased patient quality of life in long-term follow-up periods.^23^ Most cost–effectiveness studies for primary total hip arthroplasty and total knee arthroplasty use quality-adjusted life years (QALYs) as the main measure of quality of life. One-year QALYs for total knee arthroplasty and primary total hip arthroplasty were 0.768 QALYs and 0.799 QALYs, respectively, while cumulative health gains were 2.8 QALYs (primary total hip arthroplasty) and 2.3 QALYs (total knee arthroplasty) over 15 years.^24^ Total hip arthroplasty tends to be slightly more cost–effective than total knee arthroplasty, although total knee arthroplasty is more commonly performed.^25^ In 2015, the cost of scaling up surgical services globally was estimated to be US$ 300–420 billion, but costs must also be viewed in terms of their potential economic benefit when justifying future investment in surgical capacity-building, especially for elective surgeries.^26^ A recent paper argued that surgical investment may unlock a substantial prosperity dividend, as governments across all income settings seek ways to reverse disabling disease and return patients to work as a way of boosting economic productivity.^7^ Arthroplasty has a unique ability to return patients to work; a meta-analysis of 9267 patients having total hip arthroplasty found 28.1% (95% CI: 17.2–42.2) of patients who were not working before surgery began working afterwards.^27^ Furthermore, total lifetime expenditure on long-term assisted living was estimated to decrease by 28% in patients undergoing arthroplasty for end-stage hip osteoarthritis compared with patients managed without surgery.^28^

## Feasibility of implementation

As the foundation of UHC, primary health care places emphasis on population needs through comprehensive integrated health services, policies addressing upstream determinants of health and community involvement using local resources. With improved control of the communicable disease burden, and increasing lifespans in low- and middle-income countries with the consequent higher incidence of musculoskeletal conditions, expanding primary health-care services to include arthroplasty will be needed to address increased patient demand for such conditions. As patents for many successful orthopaedic surgery procedures and products start to expire, manufacturing generic implants with identical design specifications as the most bio-durable hip prostheses will likely reduce costs and improve accessibility of orthopaedic implants.^29^ A basic arthroplasty package, specifying the minimum criteria to safely perform primary hip and knee arthroplasty, should be defined to facilitate policy planning, cost projections and supply-chain planning. With raw materials available locally, harnessing local means of production will promote primary health care by increasing community involvement.

Surgical volume is large and growing, with 312.9 million total surgeries (95% CI: 266.2 million–359.5 million) performed in 2012 representing an increase from 226.4 million in 2004. The two regions with the greatest surgical need are western and eastern sub-Saharan Africa.^18^ The only systematic review of arthroplasty in sub-Saharan Africa included 12 articles comprising 606 total hip arthroplasties and 763 total knee arthroplasties; osteonecrosis was the most common indication for total hip arthroplasty, while osteoarthritis was most common for total knee arthroplasty, with an HIV prevalence in patients of up to a third.^30^ The complication profiles reported for the procedures were similar to the arthroplasty complications in high-income countries, suggesting feasibility. The estimated dislocation rate with total hip arthroplasty was 1.6% (7/444), while prosthetic joint infections were estimated to have occurred in 0.5% (2/444)of total hip arthroplasties and 1.6% (9/555)of total knee arthroplasties, although reporting bias may have affected these findings.^30^

Arthroplasty also yields predictable and durable outcomes by following standardized clinical pathways. Standardized pathway procedures make arthroplasty well-suited for large-scale worldwide implementation. Additionally, primary total hip and total knee arthroplasty practice has effectively shifted to the ambulatory surgical setting in high-income countries with same-day discharges.^31^ If these surgeries can also be done in outpatient settings in resource-constrained settings, less stress would be placed on the constrained in-patient hospital resources. Despite the potential benefits of expanding arthroplasty to manage the large disease burden by delivering cost-effective outcomes and economic benefits, such as returning patients to productive work and improving quality of life, the implementation of arthroplasty in resource-constrained settings may divert personnel, funding and infrastructure from other essential surgical procedures. This situation presents an ethical and economic trade-off that policy-makers must manage at a local level. We advocate that essential surgical procedures should not be viewed from a zero-sum perspective whereby the addition and promotion of arthroplasty reverses global surgical progress elsewhere, as surgery is a cornerstone of robust health systems that promotes public health.

To formulate, implement and finance surgical capacity-building, some low- and middle-income countries have committed to developing national surgical, obstetric and anaesthesia plans to make progress on development goals.^32^ These plans aim to scale up surgical systems countrywide through eight steps: (i) health ministry support and ownership; (ii) baseline situational assessments; (iii) stakeholder engagement for priority-setting; (iv) drafting and validation; (v) monitoring for evaluation; (vi) costing; (vii) governance; and (viii) implementation. National surgical, obstetric and anaesthesia plans are meant to define current gaps, develop solutions, and provide an implementation and monitoring plan with a projected cost for six areas of a surgical system: (i) infrastructure; (ii) service delivery; (iii) workforce; (iv) information management; (v) finance; and (vi) governance.^33^ Future national surgical, obstetric and anaesthesia plans should introduce mandatory joint registries to enable accurate monitoring in low- and middle-income countries that are scaling up arthroplasty. Notably, even in high-income countries, demand for joint arthroplasty is projected to outpace the supply of orthopaedic surgeons, with surgeon caseloads expected to double by 2050 unless proactive workforce expansion occurs.^34^ This projected lack of surgeons underscores the urgency of parallel worldwide investment in surgical training and workforce development.

## Future challenges

Surgical care is needed in the treatment of nearly all disease categories within the context of the Global Burden of Disease, which strengthens the argument that these services must be integrated into health systems at all levels and for different clinical problems.^35^ In sub-Saharan Africa, the district hospital lends itself to feasible integration of essential surgery within comprehensive primary health-care services to contribute to a sustainable reduction in preventable morbidity and mortality.^36^ However, the most important barrier to the safe provision of preoperative, intraoperative and postoperative surgical and anaesthesia services in low- and middle-income countries is the shortage of trained workers.^5^ Sub-Saharan Africa has less than 1% of the number of surgeons practising in the USA, despite having a population that is three times as large. This gap is due to the low number of medical school graduates, inadequate training, poor salaries and working conditions, and an inability to retain staff in remote and rural regions.^37^ Additionally, postoperative rehabilitation is essential to achieve the best surgical outcomes after arthroplasty. However, the World Health Organization’s (WHO) *World report on disability* in 2011 noted that data on the unmet need for rehabilitation services were sparse, but estimated that fewer than 10 skilled rehabilitation practitioners per 1 million population were available in low- and middle-income countries.^38^

Measures to expand surgical access, such as task-sharing, have been shown to be safe and effective while countries make long-term investments in building surgical and anaesthesia workforces.^5^ A systematic review of task-shifting and task-sharing to strengthen the surgical workforce in sub-Saharan Africa found that non-surgeon clinicians were primarily trained in simple general and emergency surgeries, with some having additional subspecialty training in orthopaedics for amputations and fracture manipulation.^39^ In Malawi, to tackle the lack of orthopaedic specialists, non-surgeon clinicians were trained to undertake some surgery. No differences were found in perioperative outcomes for major amputations and open reduction internal fixation of acute fractures. At the same time, this approach was highly cost-effective (US$ 92.06 per DALY averted) and helped transfer orthopaedic skills to rural areas.^40^ Arthroplasty lends itself well to practice by non-surgeon clinicians, given it has a standard, step-wise surgical approach and well-established clinical pathways. More evidence is needed to establish the limits of what tasks can be safely shifted to non-surgeon clinicians, but in the short term, practitioners’ skills have been effectively upgraded by providing targeted training in key procedures.^41^

The supply chain is also a key issue facing global orthopaedic surgery. The availability of surgical equipment is vital to allow hospitals to provide safe surgery and to retain surgical and anaesthesia staff by increasing their work satisfaction.^42^ The availability of pulse oximetry, essential medicines and key infrastructure (water, electricity and oxygen) varies widely between and within low- and middle-income countries. Of the 793 hospitals surveyed in low-income countries, 21.9% (174) operated without running water; 24.0% (190) without an oxygen supply; and 31.0% (246) without consistent electricity.^2^^,^^43^ Before any capacity-building interventions are implemented, existing surgical capacity must be assessed. Tools to make this assessment include the WHO tool for situational analysis to assess emergency and essential surgical care; and the personnel, infrastructure, procedures, equipment and supplies tool.^42^^,^^44^ To direct capacity-building efforts, policy-makers must have a basic arthroplasty package that defines the minimum criteria to safely perform primary arthroplasty procedures. The coming expiration of many patents for proven, durable arthroplasty implants will likely lead to the manufacture of generic implants with identical design specifications which will decrease cost and increase accessibility.^29^

With recent changes in the provision of foreign aid and research funding, ways to obtain capital to expand arthroplasty provision must be reconsidered. Although many global agencies are down-sizing and making cuts to adjust to changes in the availability of funding, reform is needed. Increasing domestic health investments and domestic financing amid shrinking foreign aid must be prioritized to avoid future dependency. Sustainable development assistance requires strategic, government-backed approaches. Although international short-term relief missions have contributed to immediate relief in crisis situations, these missions cannot substitute for capacity-building investment that develops independence, which have shown promise in previous global academic orthopaedic collaborations.^45^

## Conclusion

Essential surgical procedures are defined by their ability to address a large burden of disease, deliver high value through cost–effectiveness and remain feasible for implementation even in resource-constrained settings. Arthroplasty fulfils all three criteria as its well-established clinical pathways, predictable outcomes, and its use of standard surgical procedures make its implementation feasible at the global level. As musculoskeletal disorders (particularly osteoarthritis) emerge as a leading cause of global disability, especially in ageing populations in low- and middle-income countries, access to arthroplasty will become increasingly important. Integrating arthroplasty into future national surgical, obstetric and anaesthesia plans is essential to manage the growing mismatch between surgical demand and workforce capacity in countries of all income levels. Strategic investment in training, infrastructure and sustainable funding mechanisms will be crucial to ensure equitable access to arthroplasty, which transforms lives through restored mobility and function across the globe.

## References

[1] Price R, Makasa E, Hollands M. World Health Assembly Resolution WHA68.15: “Strengthening emergency and essential surgical care and anesthesia as a component of universal health coverage”—addressing the public health gaps arising from lack of safe, affordable and accessible surgical and anesthetic services. World J Surg. 2015 Sep;39(9):2115–25. 10.1007/s00268-015-3153-y 26239773

[2] Meara JG, Leather AJM, Hagander L, Alkire BC, Alonso N, Ameh EA, et al. Global Surgery 2030: evidence and solutions for achieving health, welfare, and economic development. Lancet. 2015 Aug 8;386(9993):569–624. 10.1016/S0140-6736(15)60160-X 25924834

[3] Alkire BC, Raykar NP, Shrime MG, Weiser TG, Bickler SW, Rose JA, et al. Global access to surgical care: a modelling study. Lancet Glob Health. 2015 Jun;3(6):e316–23. 10.1016/S2214-109X(15)70115-4 25926087 PMC4820251

[4] Debas HT, Donkor P, Gawande A, Jamison DT, Kruk ME, Mock CN, editors. Essential surgery: disease control priorities. Volume 1. 3rd ed. Washington, DC: The International Bank for Reconstruction and Development and The World Bank; 2015. Available from: https://www.ncbi.nlm.nih.gov/books/NBK333500/ [cited 2021 Jul 27]. 26740991

[5] Mock CN, Donkor P, Gawande A, Jamison DT, Kruk ME, Debas HT; DCP3 Essential Surgery Author Group. Essential surgery: key messages from Disease Control Priorities, 3rd edition. Lancet. 2015 May 30;385(9983):2209–19. 10.1016/S0140-6736(15)60091-5 25662414 PMC7004823

[6] Hyman GY, Obayagbona KI, Mugwe R, Makasa EM. The need for children’s surgical care prioritisation in national surgical care policies: a systematic review of national surgical obstetric and anaesthetic plans (NSOAPs) in sub-Saharan Africa. J Pediatr Surg. 2024 Feb;59(2):299–304. 10.1016/j.jpedsurg.2023.10.040 38135547

[7] Nepogodiev D, Picciochi M, Ademuyiwa A, Adisa A, Agbeko AE, Aguilera ML, et al. Surgical health policy 2025–35: strengthening essential services for tomorrow’s needs. Lancet. 2025 Aug 23;406(10505):860–80. 10.1016/S0140-6736(25)00985-7 40675172

[8] Hunter DJ, Reddy KS. Noncommunicable diseases. N Engl J Med. 2013 Oct 3;369(14):1336–43. 10.1056/NEJMra1109345 24088093

[9] Atlas of African health statistics 2022: health situation analysis of the WHO African Region. Geneva: World Health Organization; 2022. Available from: https://iris.who.int/handle/10665/364851 [cited 2025 Dec 2].

[10] Ferrari AJ, Santomauro DF, Aali A, Abate YH, Abbafati C, Abbastabar H, et al.; GBD 2021 Diseases and Injuries Collaborators. Global incidence, prevalence, years lived with disability (YLDs), disability-adjusted life-years (DALYs), and healthy life expectancy (HALE) for 371 diseases and injuries in 204 countries and territories and 811 subnational locations, 1990–2021: a systematic analysis for the Global Burden of Disease Study 2021. Lancet. 2024 May 18;403(10440):2133–61. 10.1016/S0140-6736(24)00757-8 38642570 PMC11122111

[11] Steinmetz JD, Culbreth GT, Haile LM, Rafferty Q, Lo J, Fukutaki KG, et al.; GBD 2021 Osteoarthritis Collaborators. Global, regional, and national burden of osteoarthritis, 1990-2020 and projections to 2050: a systematic analysis for the Global Burden of Disease Study 2021. Lancet Rheumatol. 2023 Aug 21;5(9):e508–22. 10.1016/S2665-9913(23)00163-7 37675071 PMC10477960

[12] McConaghy K, Klika AK, Apte SS, Erdemir A, Derwin K, Piuzzi NS. A call to action for musculoskeletal research funding: the growing economic and disease burden of musculoskeletal conditions in the United States is not reflected in musculoskeletal research funding. J Bone Joint Surg Am. 2023 Mar 15;105(6):492–8. 10.2106/JBJS.22.00693 36574617

[13] Sing CW, Lin TC, Bartholomew S, Bell JS, Bennett C, Beyene K, et al. Global epidemiology of hip fractures: secular trends in incidence rate, post-fracture treatment, and all-cause mortality. J Bone Miner Res. 2023 Aug;38(8):1064–75. 10.1002/jbmr.4821 37118993

[14] Moja L, Piatti A, Pecoraro V, Ricci C, Virgili G, Salanti G, et al. Timing matters in hip fracture surgery: patients operated within 48 hours have better outcomes. A meta-analysis and meta-regression of over 190,000 patients. PLoS One. 2012;7(10):e46175. 10.1371/journal.pone.0046175 23056256 PMC3463569

[15] Gibbs AJ, Gray B, Wallis JA, Taylor NF, Kemp JL, Hunter DJ, et al. Recommendations for the management of hip and knee osteoarthritis: a systematic review of clinical practice guidelines. Osteoarthritis Cartilage. 2023 Oct;31(10):1280–92. 10.1016/j.joca.2023.05.015 37394226

[16] Jüni P, Hari R, Rutjes AWS, Fischer R, Silletta MG, Reichenbach S, et al. Intra-articular corticosteroid for knee osteoarthritis. Cochrane Database Syst Rev. 2015 Oct 22;2015(10):CD005328. 10.1002/14651858.CD005328.pub3 26490760 PMC8884338

[17] Shrime MG, Bickler SW, Alkire BC, Mock C. Global burden of surgical disease: an estimation from the provider perspective. Lancet Glob Health. 2015 Apr 27;3 Suppl 2:S8–9. 10.1016/S2214-109X(14)70384-5 25926322

[18] Rose J, Weiser TG, Hider P, Wilson L, Gruen RL, Bickler SW. Estimated need for surgery worldwide based on prevalence of diseases: a modelling strategy for the WHO Global Health Estimate. Lancet Glob Health. 2015 Apr 27;3(Suppl 2):S13–20. 10.1016/S2214-109X(15)70087-2 25926315 PMC5746187

[19] McCord C, Chowdhury Q. A cost-effective small hospital in Bangladesh: what it can mean for emergency obstetric care. Int J Gynaecol Obstet. 2003 Apr;81(1):83–92. 10.1016/S0020-7292(03)00072-9 12676406

[20] Gosselin RA, Thind A, Bellardinelli A. Cost/DALY averted in a small hospital in Sierra Leone: what is the relative contribution of different services? World J Surg. 2006 Apr;30(4):505–11. 10.1007/s00268-005-0609-5 16528459

[21] Chao TE, Sharma K, Mandigo M, Hagander L, Resch SC, Weiser TG, et al. Cost–effectiveness of surgery and its policy implications for global health: a systematic review and analysis. Lancet Glob Health. 2014 Jun;2(6):e334–45. 10.1016/S2214-109X(14)70213-X 25103302

[22] Graham SM, Howard N, Moffat C, Lubega N, Mkandawire N, Harrison WJ. Total hip arthroplasty in a low-income country: ten-year outcomes from the national joint registry of the Malawi Orthopaedic Association. JBJS Open Access. 2019 Dec 5;4(4):e0027. 10.2106/JBJS.OA.19.00027 32043050 PMC6959913

[23] Konopka JF, Lee YY, Su EP, McLawhorn AS. Quality-adjusted life years after hip and knee arthroplasty: health-related quality of life after 12,782 joint replacements. JBJS Open Access. 2018 Aug 15;3(3):e0007. 10.2106/JBJS.OA.18.00007 30533590 PMC6242318

[24] Lan RH, Yu J, Samuel LT, Pappas MA, Brooks PJ, Kamath AF. How are we measuring cost–effectiveness in total joint arthroplasty studies? Systematic review of the literature. J Arthroplasty. 2020 Nov;35(11):3364–74. 10.1016/j.arth.2020.06.046 32680755

[25] Bumpass DB, Nunley RM. Assessing the value of a total joint replacement. Curr Rev Musculoskelet Med. 2012 Dec;5(4):274–82. 10.1007/s12178-012-9139-6 23054621 PMC3702749

[26] Verguet S, Alkire BC, Bickler SW, Lauer JA, Uribe-Leitz T, Molina G, et al. Timing and cost of scaling up surgical services in low-income and middle-income countries from 2012 to 2030: a modelling study. Lancet Glob Health. 2015 Apr 27;3 Suppl 2:S28–37. 10.1016/S2214-109X(15)70086-0 25926318

[27] Soleimani M, Babagoli M, Baghdadi S, Mirghaderi P, Fallah Y, Sheikhvatan M, et al. Return to work following primary total hip arthroplasty: a systematic review and meta-analysis. J Orthop Surg Res. 2023 Feb 12;18(1):95. 10.1186/s13018-023-03578-y 36782319 PMC9926652

[28] Kunkel ST, Sabatino MJ, Kang R, Jevsevar DS, Moschetti WE. The cost–effectiveness of total hip arthroplasty in patients 80 years of age and older. J Arthroplasty. 2018 May;33(5):1359–67. 10.1016/j.arth.2017.11.063 29276115

[29] Uzoigwe CE, Shoaib A. Patents and intellectual property in orthopaedics and arthroplasty. World J Orthop. 2020 Jan 18;11(1):1–9. 10.5312/wjo.v11.i1.1 31966964 PMC6960299

[30] Davies PS, Graham SM, Maqungo S, Harrison WJ. Total joint replacement in sub-Saharan Africa: a systematic review. Trop Doct. 2019 Apr;49(2):120–8. 10.1177/0049475518822239 30636518 PMC6535807

[31] DeCook CA. Outpatient joint arthroplasty: transitioning to the ambulatory surgery center. J Arthroplasty. 2019 Jul;34(7) 7S:S48–50. 10.1016/j.arth.2019.01.006 30773355

[32] Truché P, Shoman H, Reddy CL, Jumbam DT, Ashby J, Mazhiqi A, et al. Globalization of national surgical, obstetric and anesthesia plans: the critical link between health policy and action in global surgery. Global Health. 2020 Jan 2;16(1):1. 10.1186/s12992-019-0531-5 31898532 PMC6941290

[33] Sonderman KA, Citron I, Mukhopadhyay S, Albutt K, Taylor K, Jumbam D, et al. Framework for developing a national surgical, obstetric and anaesthesia plan. BJS Open. 2019 Jul 24;3(5):722–32. 10.1002/bjs5.50190 31592517 PMC6773655

[34] Rullán PJ, Deren ME, Zhou G, Emara AK, Klika AK, Schiltz NK, et al. The arthroplasty surgeon growth indicator: a tool for monitoring supply and demand trends in the orthopaedic surgeon workforce from 2020 to 2050. J Bone Joint Surg Am. 2023 Jul 5;105(13):1038–45. 10.2106/JBJS.22.00874 36897960

[35] Rose J, Chang DC, Weiser TG, Kassebaum NJ, Bickler SW. The role of surgery in global health: analysis of United States inpatient procedure frequency by condition using the Global Burden of Disease 2010 framework. PLoS One. 2014 Feb 26;9(2):e89693. 10.1371/journal.pone.0089693 24586967 PMC3935922

[36] Galukande M, von Schreeb J, Wladis A, Mbembati N, de Miranda H, Kruk ME, et al. Essential surgery at the district hospital: a retrospective descriptive analysis in three African countries. PLoS Med. 2010 Mar 9;7(3):e1000243. 10.1371/journal.pmed.1000243 20231871 PMC2834708

[37] Ozgediz D, Riviello R, Rogers SO. The surgical workforce crisis in Africa: a call to action. Bull Am Coll Surg. 2008 Aug;93(8):10–6. 19492736

[38] World report on disability. Geneva: World Health Organization; 2011. Available from: https://iris.who.int/handle/10665/44575 [cited 2025 Dec 2]. 10.3390/ijerph15102165

[39] Ryan I, Shah KV, Barrero CE, Uamunovandu T, Ilbawi A, Swanson J. Task shifting and task sharing to strengthen the surgical workforce in sub-Saharan Africa: a systematic review of the existing literature. World J Surg. 2023 Dec;47(12):3070–80. 10.1007/s00268-023-07197-w 37831136

[40] Wilhelm TJ, Dzimbiri K, Sembereka V, Gumeni M, Bach O, Mothes H. Task-shifting of orthopaedic surgery to non-physician clinicians in Malawi: effective and safe? Trop Doct. 2017 Oct;47(4):294–9. 10.1177/0049475517717178 28682219

[41] Shrime MG, Sleemi A, Ravilla TD. Charitable platforms in global surgery: a systematic review of their effectiveness, cost-effectiveness, sustainability, and role training. World J Surg. 2015 Jan;39(1):10–20. 10.1007/s00268-014-2516-0 24682278 PMC4179995

[42] Oosting RM, Wauben LSGL, Groen RS, Dankelman J. Equipment for essential surgical care in 9 countries across Africa: availability, barriers and need for novel design. Health Technol (Berl). 2019;9(3):269–75. 10.1007/s12553-018-0275-x

[43] LeBrun DG, Chackungal S, Chao TE, Knowlton LM, Linden AF, Notrica MR, et al. Prioritizing essential surgery and safe anesthesia for the post-2015 development agenda: operative capacities of 78 district hospitals in 7 low- and middle-income countries. Surgery. 2014 Mar;155(3):365–73. 10.1016/j.surg.2013.10.008 24439745

[44] Tool for situational analysis to assess emergency and essential surgical care. Geneva: World Health Organization; 2011. Available from: https://www.who.int/docs/default-source/integrated-health-services-%28ihs%29/csy/gieesc/whotoolsituationalanalysiseesc.pdf [cited 2025 Dec 2]. 10.1007/s00268-010-0918-1 PMC3032911 21190114

[45] Conway DJ, Coughlin R, Caldwell A, Shearer D. The Institute for Global Orthopedics and Traumatology: a model for academic collaboration in orthopedic surgery. Front Public Health. 2017 Jun 30;5:146. 10.3389/fpubh.2017.00146 28713803 PMC5491941

